# Advances on Self-Regulation Models: A New Research Agenda Through the SR vs ER Behavior Theory in Different Psychology Contexts

**DOI:** 10.3389/fpsyg.2022.861493

**Published:** 2022-07-15

**Authors:** Jesús de la Fuente, José Manuel Martínez-Vicente, Flavia H. Santos, Paul Sander, Salvatore Fadda, Evangelia Karagiannopoulou, Evely Boruchovitch, Douglas F. Kauffman

**Affiliations:** ^1^School Education and Psychology, University of Navarra, Pamplona, Spain; ^2^School of Psychology, University of Almería, Almería, Spain; ^3^School of Psychology, University College of Dublin, Dublin, Ireland; ^4^School of Psychology, Tesside University, Middlesbrough, United Kingdom; ^5^Unit of Prevention of Stress, University of Sassari, Sassari, Italy; ^6^Department of Psychology, School of Social Sciences, Institute of Humanities and Social Sciences, University Research Centre of Ioannina, Ioannina, Greece; ^7^School of Education, UNICAMP State University of Campinas, São Paulo, Brazil; ^8^School of Clinical Medicine, Medical University of the Americas–Nevis, Devens, MA, United States

**Keywords:** Albert Bandura, social cognitive theory, self-determination, self-regulation, self- vs. external regulation

## Abstract

The aim of this paper is to demonstrate how Bandura's Social Cognitive Theory (1986) influenced the development of several complementary models of the construct of Self-Regulation. Building on the foundation of Self-Determination Theory, SDT (2000), and Zimmerman's Self-Regulation Theory, SR (2001), with their assumptions, contributions, goddesses, and limitations, we come to the Self- vs. External Regulatory Theory, SR-ER (2021). Finally, we integrate recent evidence demonstrating the explanatory adequacy of the SR vs. ER model for different psychological constructions in different settings related to education, health, clinical practice and social work. Complementary, a new theoretical and empirical research agenda is presented, to continue testing the adequacy of SR vs. ER assumptions, and to better understand the behavioral variability of the different constructs studied.

## Preface

This article is dedicated to Prof. Albert E. Bandura (1925-2021), outstanding human being and one of the most influential psychologists of all time. Bandura's ground-breaking Bobo doll experiment gave rise to the field of social learning theory, later renamed social cognitive theory. The construct of self-efficacy was identified and described by Bandura. He challenged the core assertions of behaviorism and put forward his agentic theory of human behavior. A recent APA tribute (2021) to Albert Bandura summarizes highlights of his career: “Bandura was elected APA president in 1973 and encouraged our organization to pursue matters of public interest. Bandura's significant contributions to the field of psychology were recognized in 1980 with APA's Distinguished Scientific Contribution Award and in 2004 with our Award for Outstanding Lifetime Contribution to Psychology. He also received the Gold Medal Award for Distinguished Lifetime Contribution to Psychological Science from APF and the Lifetime Career Award from the International Union of Psychological Science. In 2016, he was awarded the National Medal of Science by President Barack Obama. Albert Bandura was a giant in the field, with work that influenced social, cognitive, developmental, educational, and clinical psychology. …Bandura's contribution is irreplaceable; without it, the current view of human educational and social processes would be impossible. His writings have always marked a before and after in our understanding of psychoeducational processes”.

## Introduction

Every researcher knows that there is nothing more practical than a good theory—though not every theory can be equally applicable in practice (Berkman and Wilson, 2021). Bandura's Social Cognitive Theory (Bandura, 1986, 1991, 1999, 2004a,b, 2005, 2006) addresses the process by which a person acquires knowledge, beliefs, attitudes and ways of thinking in regard to the social environment. The foundation of this theory is that learning is an agentic, cognitive process that exists and is understood within a context of family, school, work or other (Bandura, 2006). This theoretical model established several explanatory mechanisms or types of learning -essentially human- such as learning through vicarious mechanisms or through self-regulatory mechanisms, thereby questioning and expanding the prevailing vision of the day, that of learning by classical and operant conditioning.

The aim of this manuscript is to comparatively analyze three existing theoretical models in educational psychology, all of which have adopted the construct of self-regulated behavior as a core element, but have established different explanatory mechanisms to explain its role in processes of human development and learning processes. Therefore, starting from a definition of the construct itself, the different theoretical positions will be analyzed (including goodnesses and limitations), to conclude with a prospective research proposal.

## Self-Regulation Behavior

The construct of Self-Regulation (SR) is a personality-related construction (Mithaug, 1993; Boekaerts et al., 1999; Hoyle, 2010) that describes a person's ability to plan, monitor, and evaluate their own behavior (Brown, 1998; Vohs and Baumeister, 2016). Pervin (1988) study defined the classical understanding of this psychological construction. The initial conceptualization of self-regulation, situated at the molecular level of psychological analysis (de la Fuente et al., 2019a), adopts three principles:

1) SR is a variable pertaining to the subject and determined by other subject variables or factors, such as personality and metacognitive factors (Hoyle, 2010; Valikhani et al., 2020; Vega et al., 2020).
2) Contextual factors are considered indirectly, as having a more tangential role in explaining variability or defining the level of a person's behavioral regulation, whether referring to general behavior or specific, education- or health-related behavior.
3) People are assumed to have a higher or lower level of self-regulation, without attempting to define SR categories.

The plentiful previous research has documented numerous relationships with SR: personal adjustment factors are positively related (Wrosch et al., 2003); in personality factors, Conscientiousness is positively related and Neuroticism relates negatively (Guido et al., 2015; de la Fuente et al., 2020a); and SR is positively related to well-adjusted behavior in academic achievement (Blair and Raver, 2015; Bernardo et al., 2019).

## Self-Regulation in Bandura's Theory

In Bandura's social cognitive theory (Bandura, 1986), there are interactions between *personal* factors (e.g., cognitions, feelings, skills), *behavioral* factors (e.g., strategy use, help-seeking actions), and *environmental* factors (e.g., classrooms, homes, work environments), through the concept of triadic reciprocal causality, all of which affect the individual's functioning (Usher and Schunk, 2018). The personal variable of self-efficacy (self-referential beliefs about the probability of adequate performance) results from these reciprocal influences. Prior research has demonstrated that behaviors like choice of tasks, persistence, effort, and achievement are influenced by self-efficacy beliefs (Schunk and DiBenedetto, 2016). Self-efficacy in turn is modified by students' behaviors. Students observe their progress toward learning goals as they work on their tasks. For example, assignments completed is one of many progress indicators that reinforce students' sense of capability for performing, and so increase their self-efficacy for further learning (Schunk and DiBenedetto, 2016).

Research has verified these reciprocal influences between self-efficacy and environmental variables in students with *learning disabilities*, who often have *low self-efficacy for learning* (Licht and Kistner, 1986). These individuals may react to their environment based on environment-related attributes instead of their own behavioral attributes. The learner's behaviors and the learner's environment can influence each other. The environment is influencing behavior when students pay attention to the visual without giving it much thought. Student behaviors, meanwhile, can also modify the instructional environment.

According to social cognitive theory, the individual pursues a sense of *agency*, that is, the purpose and skills to intervene and take action (Bandura, 1987, 1991), accompanied by the belief that they can exert substantial control over important aspects of their life. Self-regulation and self-efficacy are pathways to experiencing a greater sense of *agency* or *agentic perspective* (Bandura, 2001). Use of self-regulatory skills increases a students' feelings of efficacy about learning and performing well; this in turn leads to increased motivation, effort, persistence, and learning. Students' perception that they are learning enhances their agency beliefs.

## Three Complementary Models of Self-Regulation Derived from Bandura's Theory

Different theoretical models have emerged from research rooted in Bandura's Social Cognitive Theory (1986). Addressing the concept of Self-Regulation (SR) either directly or indirectly, three models rise from different fields of Psychology. Summarized and presented below, they are the object of analysis in this paper (see Table 1).

**Table 1:** Summary chart of the theoretical approaches toward *Self-Regulation* analyzed in this paper.

**Theory**	**Year**	**Discipline**	**Object of study**	**Concepts/Paradigm**	**Motivation types**	**Applications**	**Limitations**
1. Social Cognitive Theory	1997	Social psychology	Person, context	Self-efficacy, Self-regulation	Intrinsic, extrinsic	Social, educational, clinical	Micro-level analysis
2. Self-Determination Theory (SDT)	1985	Developmental psych/learning	Person, context	SR (Autonomy) vs. ER (Heteronomy)	Introjected motiv /a-motivation/external motivation	Wellbeing/ psychopathology autonomy-competence	Human development process, external regulation = external dys-motivation
3. Self-Regulated Learning Theory (SRL)	2001	Psychology of learning	Person, context	Self-Regulated Learning (SRL)	SR motivation	Learning process	Molar-level processes
4. SRL vs. ERL Theory (SRL vs. ERL)	2017	Instructional psychology	Person x context	SR vs. ER Learning Processes	SRL vs. ERL motivation, regulatory	Teaching and Learning Process	Molar-level processes
5. SR vs. ER Theory (SR vs. ER)	2021	Multiple spheres of psychology	Person x context	SR vs. ER in different contexts	SR x ER Factors	Different contexts	Micro- and molecular level process analysis

## Self-Determination Theory: Encouraging the Development of Autonomy

The first model, *Self-Determination Theory (SDT)* (Deci and Ryan, 1985a,b, 2008; Ryan and Deci, 2000b, 2006, 2020a,b) is a heuristic model of human development in interaction with the environment. SDT serves to explain how human motivation is largely determined by the needs for self-determination and autonomy. The impact of this theory in research and applied practice has been unquestionable, especially in the educational sphere of special educational needs. A Google Scholar search on *self-determination* and *self-regulation* yields a total of 63,000 documents (18-Oct-2021). This proposed theoretical framework has an indirect link to Albert Bandura's model because it gives shape to an interactive, combined conception of the mechanisms of motivation and human regulation. It concurs with Bandura's model in assuming that behavior and its development can be determined both internally and externally; furthermore, it establishes the sequential process for externally regulated behavior to become internalized. Consequently, both share the construct of self-regulation as a core explanatory element, and give importance to external factors as a regulatory mechanism.

### Assumptions

SDT is a theoretical model of the molecular-molar order (de la Fuente et al., 2019a). Its focus is to explain human development and wellbeing using an explanatory philosophical paradigm that adopts the concepts of autonomous development, as opposed to heteronomous and anomic development (“autonomy” retains its primary etymological meaning of self-governance, or rule by self-control). *Heteronomy*, as the direct opposite, refers to “regulation from outside the phenomenal self, by forces experienced as alien or pressuring, be they inner impulses or demands, or external contingencies of reward and punishment” (Deci and Ryan, 1985a, p. 1562). In reaction to the external, behaviorist paradigm of twenty years ago, Self-Determination Theory is based on three essential concepts (Deci and Ryan, 1994; Deci et al., 1996; Ryan and Deci, 2017a,b, 2020a,b): (1) *Autonomy* involves initiative and ownership of one's actions. Experiences that correspond to a person's interest and value support autonomy, while external control, either by rewards or punishment, undermines autonomy. (2) *Competence* corresponds to a sense of mastery and of being able to succeed and grow. Competence is best promoted by optimal challenges, positive feedback, and growth opportunities, offered within well-structured settings. (3) *Relatedness* involves feelings of belonging and connection and is promoted by the expression of caring and respect.

This model is widely accepted and is backed by a large volume of empirical evidence (Deci and Ryan, 1985a,b,c, 2000; Deci et al., 1994, 1996; Ryan and Deci, 2017a,b, 2020a,b; Howard et al., 2022). A recent meta-analysis reported that ego-involved motives were positively related not only to persistence and performance goals, but also to indicators of well-being. By contrast, motivation driven by a desire to obtain rewards or avoid punishment was associated with decreased well-being, and there was no association with performance or persistence. Amotivation, for its part, was related to poor outcomes (Hagger and Hamilton, 2020).

### Motivational and Regulatory Style

**Figure 1:**
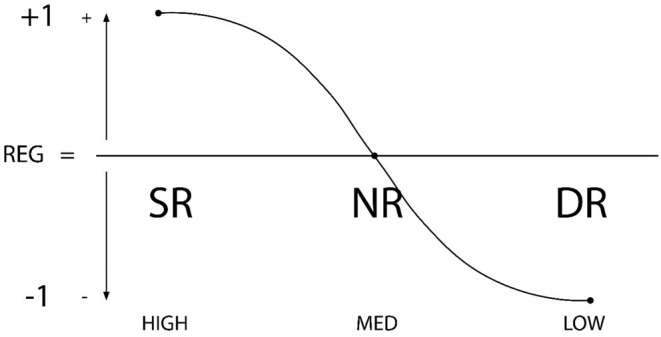
Graphic representation of regulation types: SR, Self-regulation; NR, Non-regulation; DR, Dys-Regulation. The X axis represents the degree of regulation (high-medium-low), while the Y axis shows directionality (+1, 0, −1).

Self-Determination theory has elevated the role of the student in responding to their own motivations. It conceptualizes development on the basis of personal needs, and motivation as a progressive internalizing process from external influences to internal ones, where the person constructively defines their own personal needs and motivations. The theory is based on the following assumptions: (Ryan and Deci, 2000b, p.1; see Fig. 1):

(1) There are multiple *types of motivation* with their own unique characteristic phenomenology and dynamics. The concepts of amotivation, intrinsic motivation and extrinsic motivation are taken from this theory (Ryan and Deci, 2017a,b). Types of motivation can be ordered on a self-determination continuum (Howard et al., 2017; Ryan and Deci, 2017a,b, 2020a,b), where intrinsic motivation lies on the end of high self-determination, and amotivation at the opposite end, where self-determination is absent. Partially self-determined states, such as introjection, lie between the two extremes.

(2) *Regulation styles* result from the view of *self-determination of motivation* (Howard et al., 2017), and also range from extrinsic to intrinsic. *Extrinsic regulation* stems from externally imposed rewards and punishments and is typically experienced as controlled, non-autonomous motivation. When extrinsic motivation has become partly internalized, we refer to *introjected regulation*, or regulation by internal rewards of self-esteem for success and by avoidance of anxiety, shame, or guilt for failure. In academic activities, introjected regulation often involves the ego (Deci et al., 1982); self-esteem is contingent on outcomes, resulting in “internally controlled” regulation.

(3) Attributions of outcomes and the corresponding *perceived causality* are established according to type of motivation. A meta-analytic, structural equation model revealed total effects of autonomy orientation on behavior, comprising direct and indirect effects through autonomous motivation. There was also a positive direct effect of control orientation on behavior, and a negative indirect effect through controlled motivation (Hagger and Hamilton, 2020). This motivational model has also been transferred to other fields such as health (Ntoumanis et al., 2020; Vallerand, 2021).

### Limitations

Limitations of these concepts have been recognized, in that they do not reflect a conceptual continuum, nor are they presented as complementary (not mutually exclusive). Moreover, the role of type of context as an influence in motivational processes has not been sufficiently accounted for.

(1) The authors themselves acknowledge this in their model, which emerges from the Psychology of Human Development, with extrapolations for improved learning and teaching (Ryan and Deci, 2000b). We find ample evidence and dissemination of this model in the study of special educational needs of students, including assessment and intervention (Almukhambetova and Hernández-Torrano, 2020). However, the model does not specify discrete processes of regulation of learning, nor the specific strategies of regulating motivation before, during and after the execution of a given task, as is reflected in the model by Zimmerman and Schunk (2001).

(2) The model's concept of external regulation focuses on control or application of external contingencies (a behavioral perspective) (Ryan and Deci, 2000b), and not on the possible external promotion or facilitation of the student's self-regulation. There is plentiful evidence that *external regulation*—understood in opposition to internal motivation or introjected motivation—produces poorer motivation in the behavior in question (Adams et al., 2017; Shum et al., 2021), even in the case of the COVID-19 pandemic (Morbée et al., 2021). However, research has also shown that people can operate with mixed motivational systems (de la Fuente, 2004), or changing back and forth from external to internal, according to the context (de la Fuente, 2004).

(3) The theoretical model does not incorporate a person's *regulation state or style*, which lies on a plausible continuum between self-regulated, deregulated (non-regulated), and dysregulated motivation (de la Fuente, 2017; Pachón-Basallo et al., 2021). There is no acknowledgment that a person may exhibit dysregulated behavior or motivation. However, clinical, healthcare and educational practice abound with reports showing this type of regulation to be real and pathological (Ryan et al., 2012).

(4) Also lacking is the possibility that the context may externally induce nonregulation. In fact, this aspect is yet to be defined in the theoretical model (Ryan and Deci, 2020a,b). Nor is this aspect established in the external inducement of dysregulation. Evidence has documented the existence of dysregulating contexts, in the personal and contextual realm (Pachón-Basallo et al., 2021).

### Conclusion

SDT seeks to explain and predict self-determination processes in human beings—and has done so with abundant evidence and consistency. In different teaching-learning contexts, however, such processes: (1) are insufficiently associated with specific self-regulation mechanisms that are essential to explaining autonomy and self-management behaviors in humans (Bandura, 1986; Zimmerman and Schunk, 2001); (2) underestimate the possibility that *external regulation can* actually promote *self-regulation;* in other words, external regulation is considered only in its dysregulatory version (de la Fuente, 2017); nor do they consider that a person may be intrinsically motivated or self-regulated, without needing an externalization or internalization process to become so; (3) minimize the *value of the context* in promoting self-regulation, that is, an *external regulatory value*, not understood in opposition to internal regulation nor as external control (dysregulatory), but as a promoter and aid to self-regulation (externally regulatory).

## Self-Regulated Learning Theory: Self-Regulated Learning

The theory of Self-Regulated Learning, developed by Zimmerman and Schunk (2001, 2011), offers detailed information about specific psychological processes that occur during academic/scholastic learning in reference to regulating one's own behavior. Plentiful evidence has been produced in support of this theoretical model, as well as its implications for intervening in student motivation (molecular analysis of learning). Though not addressed directly, certain principles of molar (or interaction with the context) analysis are suggested in this model. To complete this model, the processes it addresses must be incorporated within the larger, molar processes of teaching and learning. In this way, other possible types of regulation would be included along with self-regulation (Zimmerman and Labuhn, 2012).

### Assumptions

The heuristic proposed by this theory offers an orderly, systematic view of students' cognitive and motivational processes during learning (Zimmerman, 2000). Referring specifically to motivation, it offers a discrete understanding (microanalysis) of motivational and meta-motivational processes throughout the circular, recurring sequence of the learning process (Cleary et al., 2012; Reindl et al., 2020). This heuristic model, given its explanatory potential, has been expanded to other fields of human learning (White and Bembenutty, 2014), such as skill training, assessment, and intervention in health (Hennessy et al., 2020) and in sports (Balk and Englert, 2020; Taylor et al., 2020; Wolff et al., 2021).

Zimmerman (2000) expanded Bandura's vision using a three-phase cyclical model that incorporates the individual's actions before and after task performance. This allows us to see more clearly how personal, behavioral, and social/environmental factors dynamically interact. Self-regulation is thus conceived along the three phases of forethought, performance, and self-reflection:

1) Prior to performance, the *forethought phase* is when learners set goals and select strategies for meeting them. The physical and social context is also addressed in the learner's forethought phase. Materials needed for task execution are acquired, and arrangements may be made to work with others. Time management is addressed, including decisions about when, where and how to work, and the overall time to be spent on the task and its components. Learners may actively motivate themselves to work on the task. For example, they may feel self-efficacy in being capable of success, and they may remind themselves that the task is valuable or important.
2) In the performance phase, learners work on the task; they self-instructions, and observe the results of their effort along the way. They consider how well their strategies are working, and whether they are making progress toward their goal.
3) Self-reflection takes place when the task is completed, although learners may also take time out for reflection during performance. Self-reflection is the learner's evaluation of how successful they have been. They made conclude that they need a change of strategy, or to arrange better conditions for working. In light of their outcomes, they may make attributions, that is, identify what they perceive to be causes. Attributions answer the question of why one was successful or not successful. These attributions and evaluations may prompt them to keep using the same strategy or to change it.

Students with learning disabilities, by way of illustration, often have difficulty in all three phases (Schunk and DiBenedetto, 2020a). Their forethought phase may be limited, without taking the time needed to plan out goals and strategies, and they may start the task with low self-efficacy of being able to successfully carry it out. In the performance phase, they may lack focused attention on the task, not overseeing their own work or considering their progress. In self-reflection, they may not properly evaluate their performance, and they may make non-motivating attributions. If they had trouble in doing the task, for example, they may attribute this to their own lack of ability instead of less-than-adequate effort.

### Motivational and Regulatory Process

A central contribution of this model to the area of motivation is that it delimits the *self-regulation* variable at each motivational phase in cyclical learning, taking a metacognitive view, that is, becoming conscious of these processes and regulating them. This knowledge of meta-motivation or motivation regulation has been applied to many fields (Zimmerman, 2008; Monem, 2010; Panadero, 2017). At each phase of learning, the model proposes motivational behaviors that regulate the learning process:

1) *At the start of the learning activity*. The model establishes that it is possible to help students understand their own motivations and learning needs and establish learning goals, as well as plan their motivational and meta-motivational events: self-efficacy expectations (Bandura, 1987), academic behavioral confidence (Sander and de la Fuente, 2020a,b), personal improvement and achievement goals (Pintrich, 2000), and achievement emotions in anticipation of success or failure (Pekrun et al., 2014).
2) *While carrying out the learning activity*. This model has facilitated recent research for ascertaining specific behaviors of *motivation* (decisions, positive and negative emotions), and the degree of *meta-motivational control:* motivational strategies and self-instructions (Powers et al., 2020), strategies for coping with emotions (de la Fuente et al., 2017b), motivational decisions (self-reinforcement vs. self-punishment), perfectionism vs. procrastination (Garzón-Umerenkova et al., 2018).
3) *At the end of the activity*. The model establishes how self-assessment behaviors (Schunk, 1996; Zimmerman et al., 2011) and self-administration of emotions determine the final motivational state of engagement vs. burnout (de la Fuente et al., 2020e). The authors of the model establish that an adaptive evaluation supposes the recognition of errors but also a greater focus on successes. A maladaptive appraisal carries with it the self-dispensing of negative emotions. Also have causal or attributional explanations of success and failure adjusted to adjusted stability, internality, and controllability factors (Weiner, 1993).

This has represented a considerable advance in the study of regulatory processes in motivation, since it has identified concepts belonging to the meta-motivational realm, such as motivational and affective strategies, including coping strategies, which were not previously considered as belonging to models of self-regulated, academic learning, where the initial focus was on cognitive and meta-cognitive processes.

### Contributions

Research on the construct of *Self-Regulated Learning (SRL)* that is based on Social Cognitive Theory (Bandura, 2006) has been yielding plentiful empirical evidence in relation to different variables and disciplines (Bembenutty et al., 2013):

1) In the sphere of *Self-Regulated Learning* (SRL), the relationship between SRL and Self-Efficacy has been amply demonstrated. For example, we have seen the roles of self-regulation and self-efficacy in students with learning disabilities (Schunk and DiBenedetto, 2021). SRL has also demonstrated its efficacy in the aspect of university students' work at home (Bembenutty and Hayes, 2016) and in delaying gratification (Bembenutty and Karabenick, 2004).

A large part of the research has focused on explaining and applying the SRL model to specific contexts of learning (Panadero, 2017), such as mathematics (Zimmerman et al., 2011), language arts and composition in students with behavioral maladjustment (Moohr et al., 2021), and in the sciences (Peters and Kitsantas, 2010). One essential contribution has come from the study of motivational processes and their self-regulated nature (Cleary and Zimmerman, 2004; Zimmerman and Kitsantas, 2005; Pintrich and Schunk, 2006; Wolters et al., 2011). There has also been plentiful research on the role and effect of self-regulation at university, especially in relation to assigned work (Ramdass and Zimmerman, 2011). In complementary fashion, research has also addressed improved teaching and learning through classroom practices of training in self-regulation (Zimmerman and Martinez-Pons, 1986, 1990; Zimmerman, 2008; Moos and Ringdal, 2012; Bembenutty et al., 2015; White and DiBenedetto, 2015; Zimmerman et al., 2015, 2017; White and Bembenutty, 2016; White, 2017; Schunk and DiBenedetto, 2020a,b, 2021).

2) As for *SR* and the realm of Health and Healthcare, the *Self-Regulation* construct (SR) has shown very consistent relationships with clinical and health issues. In Clinical Psychology specifically, recent research has shown self-regulation to be a cross-diagnostic variable of great importance. Its importance has also been reported from the perspective of Health Psychology (Bandura, 2004a,b, 2005). Specific examples include alcohol use and risk behaviors in adolescents (Crandall et al., 2018) and the role of SR in sports (Wolff et al., 2021).

### Limitations

This model is therefore very adequate, parsimonious and powerful for assessment and intervention to train and improve motivational and meta-motivational processes, because it allows students to become aware of and put order in their cognitive-motivational processes. There is abundant evidence of intervention programs (Martínez-Vicente and de la Fuente, 2004) and the goodnesses of their application. However, the model is limited in several aspects:

1) Its explanatory domain focuses on molecular processes of learning. For this reason, it is especially adequate for training teachers and students in how to improve discrete, specific learning processes (Lombaerts et al., 2009). Specific meta-cognitive, meta-motivational and meta-emotional behavioral training is an example of the power of this model.
2) While the model can be considered to fall within the sphere of the psychology of learning, in the university context (Cassidy, 2011), it does not address in sufficient depth the role played by instructional processes, or by teaching in formal contexts. This approach would be characteristic of the domain of instructional psychology.
3) The concept of self-regulated learning does not take into account the specific concepts of deregulation (non-regulation) or dysregulation, as necessary types for explaining other, inadequate modalities of academic learning.
4) The SRL model is very focused on self-regulated, cyclical processes at the molecular level. It does not consider, however, the connection to self-regulation (SR) as a presage, personality variable in self-regulated learning (SRL), or the connection to aspects at the molar level, i.e., external regulatory processes from the context, as in regulatory teaching (de la Fuente, 2017). These limitations have prompted the development of the following theory, presented below.

## SR vs. ER Theory: Self. vs. External- Regulation Behavior in Different Contexts

The General Model developed from *SR* vs. *ER Theory* (de la Fuente et al., 2020e) takes a molar-level approach to motivational analysis (de la Fuente et al., 2019a). It is an extrapolation of the Theory of Self-Regulated vs. Externally Regulated Learning, SRL vs. ERL (de la Fuente et al., 2013a,b, 2015, 2017a,b, 2019a,b, 2020a,b,c,d,e,f,g; de la Fuente, 2017), into different behavioral contexts. In the case of SRL vs. ERL, this analysis is contextualized within the processes of scholastic teaching and learning. With respect to their own learning, students may adopt self-regulation (as in Zimmerman's model), non-regulation, or dysregulation. The students' context (in interaction with the students' personal regulation type) may be externally regulating, externally non-regulating, or externally dysregulating. Motivational processes may then be contextualized within this new theoretical framework.

### Assumptions

**Figure 2:**
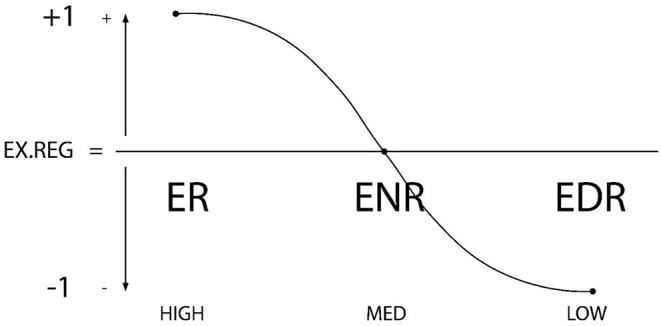
Graphic representation of external regulation types: ER, External Regulation; ENR, External Non-regulation; EDR, External Dys-Regulation. The X axis represents the degree of external regulation (high-medium-low), while the Y axis shows the directionality of the external regulation (+1, 0, −1).

*SR vs. ER* Theory (de la Fuente et al., 2020e) seeks to explain the combination of external and internal conditions that predispose adequate behavior and motivation, in response to situations in different contexts. In summary, it proposes the following:

1) An individual's competence level in *Self-Regulation* may be classified as one of three options [3 = high (self-regulation or proactive self-regulation), 2 = medium (cessation of regulation or reactive regulation); 1 = low (dysregulation or dysfunctional regulation)]. Prior research shows that the level of self-regulation that a student exercises is an indicator of their competence in self-regulation, as a personal characteristic. It also correlates to competence and adequate use of meta-motivation, meta-emotion, and meta-behavior skills (de la Fuente et al., 2015, 2017a,b). Consequently, it would also be a good indicator of *self-regulated learning* (de la Fuente et al., 2017a,b). Numeric values are assigned across the range from a higher level of personal regulation, level 3, which is the most proactive self-regulation; to a medium or non-regulatory level 2, which is not proactive; to the lowest level of self-regulation (1), or the practice of dysregulation (procrastination behavior, etc.). See Figure 1.
2) Interpersonal contexts offer *external regulation* that can also be classified across three levels [3 = high (highly externally regulatory context); 2 = medium (or external de-regulatory or non-regulating context); 1 = low (dysregulating context or external dysfunctional context). This contextual level of external regulation identifies whether the context encourages or discourages use of oversight competencies like meta-motivation, meta-emotion and meta-behavior (de la Fuente et al., 2019a,b, 2020a,b,c,d,e,f,g). Consequently, high levels in this construct indicate an *effective* or *regulatory context*. Numeric values represent a range, from a context that more effectively facilitates personal regulation, Level 3, the most proactive in promoting self-regulation; to a medium or deregulatory Level (2), offering no external support for regulation; to the lowest level of external regulation, Level 1, or external dysregulation (e.g., the teaching process triggers stress, negative achievement emotions, surface learning approaches). See Figure 2.
3) By combining these two factors we may calculate an *interactive regulation index*, between 1 and 3, that is, the average of the two regulation types, with 5 possible results (de la Fuente et al., 2019a,b, 2020a,b,c,d,e,f,g). The proposed five-combination heuristic makes it possible to analyze the most common scenarios in the interactive regulation of learning behaviors. For example, if a student is low in self-regulation (1 point), and external regulation from the context is medium (2 points), the resulting *regulation average* will be 1.5 points (2 + 1 = 3/2 = 1.5 point average); likewise, if the student has a medium level of self-regulation (2 points), but the context is low in regulation (1 point), the same regulation average is produced (2 + 1 = 3/2 = 1.5 point average). Another example might be a student who is high in self-regulation (3 points), but their context is low in regulation (1 point); the regulation average will be 2 points (3 + 1 = 4/2 = 2 points). Regulation averages can thus be ordered across a *regulation range* where the person-context interaction progresses from least favorable to most favorable: from a minimum average of 1 point (1-point personal self-regulation and 1-point external regulation), to a maximum of 3 points (3-point self-regulation and 3-point external regulation). The possible regulation averages can then be ranked in order from 1 to 5, across the regulation range (regulation average of 1 = rank 1; regulation average of 1.5 = rank 2; regulation average of 2 = rank 3; regulation average of 2.5 = rank 4; regulation average of 3 = rank 5). See Table 2.

**Table 2:** Combinations of model parameters hypothesized by SR vs. ER Theory (de la Fuente, 2017, 2021a,b).

***Combinations of levels***				***Regulation tendency***	**Stress**	**Stress**
**SR level (range)**	**ER level (range)**	**Avg**.	**Rank**		**Protection**	**Risk**
3 (3.85–5.00) H	3 (2.84–5.00) H	3	5	High-High: High-Regulation	High protection	Low risk
2 (3.10–3.84) M	3 (2.84–5.00) H	2.5	4	Medium-High: Regulation	M-H protection	M-L risk
3 (3.85–5.00) H	2 (2.35–2.83) M	2.5	4	High-Medium: Regulation	M-H protection	M-L risk
2 (3.10–3.84) M	2 (2.35–2.83) M	2	3	Medium: Non-Regulation	Medium protection	M risk
2 (3.10–3.84) M	1 (1.00–2.34) L	1.5	2	Medium-Low: Dysregulation	M-L protection	M-H risk
1 (1.00–3.09) L	2 (2.35–2.83) M	1.5	2	Low-Medium: Dysregulation	M-L protection	M-H risk
1 (1.00–3.09) L	1 (1.00–2.34) L	1	1	Low-Low: High Dysregulation	Low protection	High risk

*L, Low; M, Medium; H, High. Effects analyzed in this investigation. See previous research reports to analyze differences (de la Fuente et al., 2019a,b, p. 12; de la de la Fuente et al., 2020a,b,c,d,e,f,g, p. 5)*.

### Motivational and Self-Regulation Concepts

Recent research has provided evidence of the value of this heuristic for determining the level of different motivational-affective variables in university students, as variables dependent on the student's level of self-regulation and the teacher's external regulation. Recent research reports have shown that the combination of the two factors (SR vs. ER) determine the more cognitive-strategic factors of motivation in university learning, that is, the student's learning approach. Thus, Rank 5 involves the highest level of deep approach (deep motivation and deep strategy), while Rank 1 represents a higher level of surface learning (surface motivation and surface strategy) (de la Fuente et al., 2017a, 2020c). In the same way, motivational-affective factors are also determined by these combination levels.

The heuristic levels presented in this study have proven to be a determining factor in many aspects, such as types of achievement emotions (de la Fuente et al., 2020a); perceived level of stress factors and symptoms in the teaching/learning process (de la Fuente et al., 2020b); coping strategies used to manage this stress (de la Fuente et al., 2020c); and attitudinal factors of motivation, such as academic behavioral confidence and procrastination (de la Fuente et al., 2020d). In all cases, Combination Rank 1 proves to be the most harmful: more negative emotions; higher levels of academic stress in factors and symptoms; more emotion-focused coping strategies, to the detriment of problem-focused strategies; lower academic behavioral confidence; and greater procrastination. By contrast, Combination Rank 5 proves to be the most desirable: more positive emotions; lower levels of academic stress factors and symptoms; more problem-focused coping strategies, without renouncing certain positive emotions; more academic behavioral confidence and less procrastination.

### Limitations

This theoretical model also has certain limitations that must be addressed. On one hand, although levels of self-regulation (1 = low; 2 = medium; 3 = high) and external regulation (1 = low; 2 = medium; 3 = high) are both highly consistent constructs, assessed by two consolidated instruments, (1) the Short Self-Regulation Questionnaire (Pichardo et al., 2014) and (2) the *Interactive Assessment of the Teaching and Learning Process, IATLP* (de la Fuente et al., 2012), measurement of variables should be improved. In fact, new instruments of SR vs. ER Theory (de la Fuente, 2022; see Appendix I) have been developed for application in the spheres of education, clinical practice and ICT use, and are able to more accurately assess the constructs of self-regulation, non-regulation and dysregulation, as conceived in the present theory. Recent research findings are encouraging.

## A Research Agenda For SR VS. ER Theory: Practical Applicability in Different Psychological Contexts

This manuscript has presented specific strategies for improving student self-regulation: (1) increasing introjected motivation and self-regulation, from the model of *Self-Determination Theory*, (2) increasing the student's level of self-regulation, adopting many principles from the *Zimmerman* cyclic model; (3) making changes in the type of personal, internal regulation that is affecting students' motivation (whether regulatory, deregulatory, or dysregulatory), following certain principles from the Self- vs. External- Regulation model; (4) increasing the teacher's level of external regulation in the classroom; (5) making changes in the type of external regulation that is affecting students' motivation (whether self-regulatory, de-regulatory, or dys-regulatory). Albert Bandura's *Social Cognitive Theory*, in conjunction with the two subsequent models, has been foundational to *SR* vs. *ER Theory* (de la Fuente, 2017).

This more recent theory faces numerous challenges. On one hand, there is the need for evidence that the assessment instrument is consistently associated with self-regulation in different languages and different populations (de la Fuente, 2022; see Annex I). Analyses performed to date have shown consistency and validity (Pachón-Basallo et al., 2021). On the other hand, it is very important to verify that this heuristic—on molecular and molar levels—is applicable and accounts for the variability in different behavioral constructs, in the main fields of Psychology and Psychiatry (Romer et al., 2021). This psychological model will allow a crossed and interactive analysis of the different personal self-regulation profiles of people, in interaction with the external regulatory characteristics of the contexts in which they operate. This is a general task of psychology, as a science and as a profession. historically excessively focused on explaining and making predictions only from the individual characteristics of the subjects. Our own previous research has documented the effect of levels of self-regulation and external regulation on different types of variables and contexts (see Table 3):

1) In the sphere of *Educational Psychology*, recent research has contributed evidence of the different effects of combined levels of Self- vs. External- Regulation (SR-ER) in education. Specifically, a combined effect has been observed in learning approaches (de la Fuente et al., 2017a, 2019a; de la Fuente et al., 2020a,f), academic emotions (de la Fuente et al., 2019a,b) academic confidence and procrastination (Sander and de la Fuente, 2020a,b; de la Fuente et al., 2021c), coping strategies for academic stress (de la Fuente et al., 2017a); levels of engagement-burnout (de la Fuente et al., 2020e), positivity, resilience (de la Fuente et al., 2021d), stress factors and symptoms (de la Fuente et al., 2021a). These results were initially obtained by combining measurements from the Self-Regulation Scale (Pichardo et al., 2014; Garzón-Umerenkova et al., 2017) and the IATLP Scales (de la Fuente and Martínez-Vicente, 2008) and later using the *Self- vs. External- Regulation of Learning Inventory* (de la Fuente, 2022).
2) In the sphere of *Developmental Psychology*, this theoretical model enables us to understand the different processes of human development that depend on or are associated with levels of behavioral regulation at each stage of development, the role of regulatory characteristics of the context, and how these interact. Recent evidence has established this relationship by more deeply exploring the role of a regulatory or dysregulatory family context and its effect on learning and achievement (Balaguer et al., 2021), as well as the sometimes dysregulatory role of the social/family context in young-adult university students, in maturational disorders typical of executive dysfunction and emotional dysregulation (de la Fuente et al., 2022).
3) In the sphere of *Clinical and Health Psychology*, there is also evidence of the degree to which the SR-ER combination can predict variables like procrastination and health (Pachón-Basallo et al., 2021). The scale used in this case is the *Self- vs. External- Regulation of Learning Scale* (de la Fuente et al., 2020c). SR vs. ER theory has also been applied to analysis and behavioral prevention in the COVID-19 pandemic (de la Fuente et al., 2021e). In the same line as our results, there is documented evidence in relation to the important regulatory role of parents via modeling and the design of the behavioral context (Callejas et al., 2021). Nonetheless, the effects of these cross-diagnostic variables (SR vs. ER) is yet to be analyzed in other areas of the field of psychology:
4) In the area of *Social and Organizational Psychology*, these assumptions must be tested. The relationship should be established between the proposed SR vs. ER heuristic and specific variables of the social and organizational spheres, such as organizational engagement-burnout, psychological wellbeing in organizations, and levels of performance supported by the organizations themselves.
5) In the area of *Traffic Psychology*, the ability of the proposed heuristic to explain the behavioral variability of drivers and accident rates should be analyzed. It seems plausible to expect this explanatory ability, given that the “road trip metaphor” (de la Fuente, 2004, based on Pintrich, 1991) is what gave rise to the SR vs. ER theory. The effect of the heuristic combination in determining the level of the behavioral variables associated with driving must be demonstrated.
6) In the field of *Moral Psychology*, there is also a need to establish the connections between the SR vs. ER heuristic and issues inherent to this field, such as character strengths, spirituality, and others (Villacís et al., 2021). It is necessary to advance in the study of moral behavior (Nucci, 2014), based on the knowledge of the regulatory, personal and contextual factors, in interaction. For this, this heuristic and its instruments are a new opportunity to approach.

**Table 3:** Summarized research agenda for *Self-* vs. *External-Regulation Theory* (*SR* vs. *ER Theory)*, applied to different fields of the study of behavior in different contexts.

**Self-regulation**	**Non-regulation**	**Dys-regulation**	**Construct**	**Research**
**1. Educational psychology area (Self- vs. External-Regulated Learning Theory; SRL vs. ERL Theory)**				
**Individual variables**				
Self-regulation	Non-Regulation /Fatigue	Dys-regulation	Self-Regulation Behavior	de la Fuente, 2017; de la Fuente et al., 2021c
Self-regulated learning	Non-regulated learning	Dys-regulated Learning	Self-regulated learning	de la Fuente, 2017; de la Fuente et al., 2017a,b;
Self-control of study	Depletion toward study	Dys-control of Study	Self-control of study	Amate-Romera and de la Fuente, 2021
Problem focused coping	Emotion focused (+)	Emotion focused (−)	Academic coping strategies	de la Fuente et al., 2020d
Self-regulation (non procrastination)	Passive procrastination	Active procrastination	Procrastination	Garzón-Umerenkova et al., 2018; de la Fuente et al., 2020a
Self-motivation	Self non-motivation	Self-dysmotivation	Self-handicapping	Núñez et al., 2020
Non-anxiety	Mixed	Test-anxiety	Test anxiety	de la Fuente et al., 2017a; Amate-Romera and de la Fuente, 2021
Deep approaches	Mixed	Surface approaches	Learning approaches	de la Fuente et al., 2008, 2017a, 2020a,f
Engagement	Mixed	Burnout	Engagement-Burnout	de la Fuente et al., 2021d
Resilience high	Resilience medium	Resilience low	Resilience	
Achievement emotions (+)	Mixed achiev. emotions (=)	Achievement emotions (-)	Achievement emotions	de la Fuente et al., 2017a, 2020b
Competitive	Hard-working, impatience	Hostility/impatience	Action-emotion style	de la Fuente et al., 2016, 2017a
No stress	Distress	High Stress	Academic Stress	de la Fuente et al., 2020c
Confidence	No confidence	Dys-confidence	Academic Confidence	de la Fuente et al., 2017a, 2021a; Sander and de la Fuente, 2020a,b
High	Medium	Low	Learning achievement	de la Fuente et al., 2017a, 2020f
Strengths	Medium	Weaknesses	Character strengths	de la Fuente et al., in review
**Ext. Regulation**	**Ext. Non-regulation**	**Ext. Dys-regulation**	**Construct**	**Research**
**Contextual variables**				
External regulatory teaching	External non-regulatory teaching	External dys-regulatory teaching	Regulatory teaching	de la Fuente et al., 2017a,b, 2021a
Low (low factors)	Medium (medium factors)	High (high factors)	Contextual stress factors	de la Fuente et al., 2021a
Parental involvement	Parental non involvement	Parental dys- involvement	Parental involvement	Sander et al., 2021
Authoritative style	Permissive style (laisser-faire)	Authoritarian style	Family style	Balaguer et al., 2021
**Self-regulation**	**Non-regulation**	**Dys-regulation**	**Construct**	**Research**
**2. Health psychology area (Self- vs. External-Regulated Health Behavior Theory; SRH vs. ERH Theory)**				
**Individual variables**				
Self-regulation health	Non-regulation health/fatigue	Dys-regulation health	Self-regulation health	de la Fuente, 2017; de la Fuente et al., 2021e
Problem focused (+) coping	Emotion focused (+)	Emotion focused (−)	Coping strategies	de la Fuente et al., 2020e,g
Engagement	Mixed level medium	Burnout	Engagement-burnout	de la Fuente et al., 2020e, 2021d
Acceptance of norms	Non-acceptance of norms	Reactance to norms	Psychological reactance	Pachón-Basallo et al., 2021
Resilience	Non-resilience	Weakness	Resilience	de la Fuente et al., 2017b
Positivity	Mixed	Negativity	Positivity-negativity	de la Fuente et al., 2021d
Flourishing	Non-flourishing		Flourishing	Garzón-Umerenkova et al., 2020
Well-being (high)	Mixed (medium)	Discomfort (low)	Well-being	Becerra and Campitelli, 2013; López-Madrigal et al., 2021; de la Fuente et al., in review
Strengths	Medium	Weaknesses	Character strengths	de la Fuente et al., in review, ……
High adaptability	Medium adaptability	Dys-adaptability (low)	Adaptability	
**Contextual variables**				
External regulation of health	External non-regulation of health	External dys-regulation of health	Regulatory health context	de la Fuente et al., in review
Low (low factors)	Medium (medium factors)	High (high factors)	Contextual stress factors	de la Fuente et al., 2021b
Authoritative style	Laisser faire	Authoritarian style	Family	
**Self-regulation**	**Non-regulation**	**Dys-regulation**	**Construct**	**Research**
**3. Clinical psychology area (Self- vs. External-Regulated Behavior Theory; SR vs. ER Theory)**				
**Individual variables**				
Self-regulation	Non-regulation /fatigue	Dys-regulation	Self-regulation behavior	de la Fuente, 2017; de la Fuente et al., 2021e
Self-control of behavior	Depletion	Dys-control of behavior	Self-control of behavior	
Conscientiousness	Extraversion, openness	Agreeableness, neuroticism	Personality	Sander and de la Fuente, 2020a,b; de la Fuente et al., 2021f
Engagement	Mixed	Burnout	Engagement-burnout	de la Fuente et al., 2021d
Acceptance norms	Non- acceptance norms	Reactance norms	Psychological reactance	de la Fuente et al., 2021a
Resilience	Non-resilience	Weakness	Resilience	de la Fuente et al., 2017b
Positivity	Mixed	Negativity	Positivity-negativity	de la Fuente et al., 2021d
Self-knowledge	Self-criticism		Depression	Kopala-Sibley and Zuroff, 2020
Perfectionistic strivings	Medium	Perfectionistic concerns	Perfectionism	Frost and Marten, 1990; Stöber, 1998; Madigan, 2019; de la Fuente et al., 2020e
Low emotional reactivity	Medium emotional reactivity	High emotional reactivity	Emotional reactivity	Becerra and Campitelli, 2013
Executive functions	De- executive function	Dys-executive function	Executive functions	de la Fuente et al., in review
Character strengths (high)	(medium)	(low)	Character strengths	Seligman and Peterson, 2004
Well-being (high)	Mixed (medium)	Discomfort (low)	Psychological well-being	Becerra and Campitelli, 2013; de la Fuente et al., in review
Self-regulation of ict use	Non-regulation ict /fatigue	Dys-regulation ict	Self-regulation ict	de la Fuente, 2017; Romer et al., 2021
Self-assessment	Self-avoidance	Self-rumination	Self-assessment	
High adaptability	Medium adaptability	Dys-adaptability (low)	Adaptability	
**External regulation**	**External non-regulation**	**External dys-regulation**	**Construct**	**Research**
**Contextual variables**				
External regulation of ict use	External non- regulation of ICTS		External Dys- Regulation of ICTs	Regulation of ICT use
Low (low factors)	Medium (medium factors)	High (high factors)	Contextual Stress Factors	de la Fuente et al., 2021a
**Self-regulation**	**Non-regulation**	**Dys-regulation**	**Construct**	**Research**
**4. Social Psychology Area (Self- vs. External-Regulated Social Behavior Theory; SR vs. ER Social Theory)**				
**Individual variables**				
Social self-regulation	Social non-regulation /fatigue	Social dys-regulation	Social self-regulation	de la Fuente, 2017
Competitive	Hard-working, impatience	Hostility/impatience	Action-emotion style	de la Fuente et al., 2016, 2017a
Well-being (high)	Mixed (medium)	Discomfort (low)	Psychological well-being	Becerra and Campitelli, 2013
Engagement	Mixed	Burnout	Engagement-burnout	
Assertiveness	Non-regulation	Aggressivity / inhibition	Social abilities	
Strengths	Medium	Weaknesses	Character strengths	de la Fuente et al., in review
**External regulation**	**External non-regulation**	**External dys-regulation**	**Construct**	**Research**
**Contextual variables**				
External social regulation	External social non-regulation	External social dys-regulation	Social regulation	de la Fuente et al., in review
Low (low factors)	Medium (medium factors)	High (high factors)	Contextual stress factors	de la Fuente et al., 2021a
External organizational regulation	External organizational non-regulation	External organizational dys-regulation	Organizational regulation	de la Fuente, 2017
Authoritative style	Laisser faire style	Authoritarian style	Family	de la Fuente et al., 2021b
**Self-regulation**	**Non-regulation**	**Dys-regulation**	**Construct**	**Research**
**5. Traffic psychology area (self- vs. External- regulation of traffic behavior theory; sr vs. Er traffic theory)**				
**Individual variables**				
Self-regulation	Non-regulation /fatigue	Dys-regulation	Self-regulated behavior	de la Fuente, 2017, 2021a,b; de la Fuente et al., 2021a,b,c,d,e,f
Self-control of behavior	Depletion	Dys-control of behavior	Self-control of behavior	
Competitive	Hard-working, impatience	Hostility/impatience	Action-emotion style	de la Fuente et al., 2016, 2017a
Strengths	Medium	Weaknesses	Character strengths	de la Fuente et al., in review
**External regulation**	**External non-regulation**	**External dys-regulation**	**Construct**	**Research**
**Contextual variables**				
Low (low factors)	Medium (medium factors)	High (high factors)	Contextual stress factors	de la Fuente et al., 2021a
**Self-regulation**	**Non-regulation**	**Dys-regulation**	**Construct**	**Research**
**6. Moral psychology area (self- vs. External- regulation of moral behavior theory; moral sr vs. Er theory)**				
**Individual variables**				
Self-regulation	Non-regulation /fatigue	Dys-regulation	Self-regulation behavior	de la Fuente, 2017; de la Fuente et al., 2021e
Self-control behavior	Depletion behavior	Dys-control behavior	Self-control	Behavior
Strengths	Medium	Weaknesses	Character strengths	Villacís et al., 2021
High	Medium	Low	Spirituality	
**External regulation**	**External non-regulation**	**External dys-regulation**	**Construct**	**Research**
**Contextual variables**				
Low (low factors)	Medium (medium factors)	High (high factors)	Stress factors	de la Fuente et al., 2021a

## Conclusion

Although limited in that most evidence to date has been produced with university-age youths and in an academic context, the consistency of the relationships found encourages us to continue in this line of research. Further evidence in these different fields of Psychology will allow us to affirm with greater assurance the plausibility of the SR vs. ER postulates, especially in differentiating it from the previous theories presented. The results from empirical data that we continue to collect will allow us to conclude the applicability of these postulates to the fields of Educational Psychology, Clinical and Health Psychology, Social Psychology, Traffic Psychology and Moral Psychology.

More than ever, it is time to acknowledge and thank Prof. Albert Bandura for his proposition of the self-regulatory mechanism in human beings. His model fascinated us and has inspired us to take it thus far. These results, in good measure, also belong to him. Thank you, Professor Bandura! RIP.

## Author Contributions

JF: initial conceptualization and initial writing. JM-V support for R&D projects. FS, PS, SF, AK, EB, and DK: final reviewer and writing process. All authors contributed to the article and approved the submitted version.

## Funding

This study was funded by R&D Project PGC2018-094672-B-I00, University of Navarra, Ministry of Education and Science (Spain), and the European Social Fund (EU); R and D Project UAL18- SEJ-DO31-A-FEDER. University of Almería (Spain), and the European Social Fund (EU) (www.inetas.net).

## Conflict of Interest

The authors declare that the research was conducted in the absence of any commercial or financial relationships that could be construed as a potential conflict of interest.

## Publisher's Note

All claims expressed in this article are solely those of the authors and do not necessarily represent those of their affiliated organizations, or those of the publisher, the editors and the reviewers. Any product that may be evaluated in this article, or claim that may be made by its manufacturer, is not guaranteed or endorsed by the publisher.
