# Corrigendum: Advances on self-regulation models: A new research agenda through the SR vs ER behavior theory in different psychology contexts

**DOI:** 10.3389/fpsyg.2023.1166478

**Published:** 2023-03-08

**Authors:** Jesús de la Fuente, José Manuel Martínez-Vicente, Flavia H. Santos, Paul Sander, Salvatore Fadda, Evangelia Karagiannopoulou, Evely Boruchovitch, Douglas F. Kauffman

**Affiliations:** ^1^School Education and Psychology, University of Navarra, Pamplona, Spain; ^2^School of Psychology, University of Almería, Almería, Spain; ^3^School of Psychology, University College of Dublin, Dublin, Ireland; ^4^School of Psychology, Tesside University, Middlesbrough, United Kingdom; ^5^Unit of Prevention of Stress, University of Sassari, Sassari, Italy; ^6^Department of Psychology, School of Social Sciences, Institute of Humanities and Social Sciences, University Research Centre of Ioannina, Ioannina, Greece; ^7^School of Education, UNICAMP State University of Campinas, São Paulo, Brazil; ^8^School of Clinical Medicine, Medical University of the Americas–Nevis, Devens, MA, United States

**Keywords:** Albert Bandura, social cognitive theory, self-determination, self-regulation, self- vs. external regulation

In the published article, an author name was incorrectly written as “Angélica Karagiannopoulou.” The correct spelling is “Evangelia Karagiannopoulou.” All other relevant parts of the article have been updated to reflect this amendment.

Additionally, in the published article, the reference for “de la Fuente et al., 2022” was incorrectly written as “de la Fuente, J., Martínez-Vicente, J. M., Santos, F. H., Sander, P., Fadda, S., Karagiannopoulou, A., Boruchovitch, E., and Kauffman, D. F. (2022) Advances on self-regulation models: A new research agenda through the SR vs ER behavior theory in different psychology contexts. *Front. Psychol*. 13:861493. doi: 10.3389/fpsyg.2022.861493.”

It should be “de la Fuente, J., Martínez-Vicente, J. M., Santos, F. H., Sander, P., Fadda, S., Karagiannopoulou, E., Boruchovitch, E., and Kauffman, D. F. (2022) Advances on self-regulation models: A new research agenda through the SR vs ER behavior theory in different psychology contexts. *Front. Psychol*. 13:861493. doi: 10.3389/fpsyg.2022.861493.”

The authors apologize for these errors and state that they do not change the scientific conclusions of the article in any way. The original article has been updated.

