# Supplementary material for: Advances on Self-Regulation Models: A New Research Agenda Through the SR vs ER Behavior Theory in Different Psychology Contexts
Source: Front Psychol. 2022 Jul 15;13:861493. doi: 10.3389/fpsyg.2022.861493 (PMC9336543; doi:10.3389/fpsyg.2022.861493)
Supplement: Supplementary file 1 [file Data_Sheet_1.docx]

**APPENDIX I**. **Self- vs External- Regulation Learning Behavior Inventory in the Educational Psychology Context** (de la Fuente, J., 2022). Intellectual property registration nº 765-688472 (2022/02/07)

______________________________________________________________________

1. I think consciously about my needs related to learning and academic achievement.

Options: 1 2 3 4 5

2. I plan my behavior, setting goals and objectives for learning and achievement.

Options: 1 2 3 4 5

3. I make decisions in order to achieve positive changes in my learning and study behaviors.

Options: 1 2 3 4 5

4. I observe and keep track of myself in order to see whether I am meeting my objectives in learning, study and achievement.

Options: 1 2 3 4 5

5. I self-evaluate and reflect on improvements I've made in my learning, study and achievement behaviors.

Options: 1 2 3 4 5

6. I learn from mistakes what aspects to improve in my learning, study and achievement, for future occasions.

Options: 1 2 3 4 5

7. I seldom think about my present state and needs pertaining to learning and academic achievement.

Options: 1 2 3 4 5

8. I feel that with time I'll be improving my behavior pertaining to learning, study and academic achievement.

Options: 1 2 3 4 5

9. It’s not necessary to make decisions in order to achieve changes in my learning and study behaviors,

Options: 1 2 3 4 5

10. I see changes in my learning and study behaviors coming along the way, without giving it too much attention

Options: 1 2 3 4 5

11. I don’t do anything special to produce changes in my learning and study behavior, since those will come along on their own.

Options: 1 2 3 4 5

12. I sometimes tend to make the same mistakes several times and it looks like I don’t learn from my experience when I underachieve.

Options: 1 2 3 4 5

13. I know just what to do to enjoy myself to the fullest, but not so much for learning, studying and achieving.

Options: 1 2 3 4 5

14. I plan my learning and study behavior to get around restrictions, since they seem excessive to me.

Options: 1 2 3 4 5

15. I make choices for having the most fun, even at the expense of my learning, study and achievement goals.

Options: 1 2 3 4 5

16. I prefer to exercise control for having a good time and enjoying myself, rather than use control for meeting my learning and study obligations.

Options: 1 2 3 4 5

17. My self-assessment of my behavior looks mainly at all that I have enjoyed at each moment, without focusing on mistakes in learning and study.

Options: 1 2 3 4 5

18. It doesn’t make sense in life to change your learning and study behavior, if that takes away from your enjoyment and satisfaction.

Options: 1 2 3 4 5

19. The social context that I live in (family, environment, friends) helps me become aware of my needs in learning, study and achievement.

Options: 1 2 3 4 5

20. The social context that I live in (family, environment, friends) helps me plan my behavior by setting goals and objectives for learning, study and achievement.

Options: 1 2 3 4 5

21. The social context that I live in (family, environment, friends) helps me make decisions in order to achieve positive changes in my learning, study and achievement behaviors.

Options: 1 2 3 4 5

22. The social context that I live in (family, environment, friends) helps me observe and keep track of myself so I can see whether I am meeting my objectives in learning, study and achievement.

Options: 1 2 3 4 5

23. The social context that I live in (family, environment, friends) helps me to self-evaluate and reflect on improvements that I have made in learning, study and achievement behavior.

Options: 1 2 3 4 5

24. The social context that I live in (family, environment, friends) helps me learn from my mistakes for future occasions and to improve my behavior in learning, study and achievement.

Options: 1 2 3 4 5

25. The social context that I live in (family, environment, friends) seldom refers to my behavior or needs for improvement in learning, study and academic achievement.

Options: 1 2 3 4 5

26. The social context that I live in (family, environment, friends) feels that I'll be improving my learning and study behavior with time. That’s why they don’t interfere much in my life.

Options: 1 2 3 4 5

27. The social context that I live in (family, environment, friends) gives me the idea that you don’t need to make specific decisions in order to achieve changes in your learning and study behaviors. These changes happen by themselves over time.

Options: 1 2 3 4 5

28. The social context that I live in (family, environment, friends) lets changes in learning and study behavior come about along the way, without giving it too much attention.

Options: 1 2 3 4 5

29. The social context that I live in (family, environment, friends) doesn’t do anything special to produce changes or improvements in my learning and study behavior, since those will come along on their own, with time.

Options: 1 2 3 4 5

30. The social context that I live in (family, environment, friends) allows me to make the same mistakes in learning, study and achievement several times, even if it looks like I don’t learn from experience.

Options: 1 2 3 4 5

31. The social context that I live in (family, environment, friends) encourages me to live in the present and not think too much about my own behavior in learning, study and achievement. It isn’t that important.

Options: 1 2 3 4 5

32.The social context that I live in (family, environment, friends) encourages me to plan behaviors for having fun and enjoying myself, without thinking about learning and study restrictions that limit me.

Options: 1 2 3 4 5

33. The social context that I live in (family, environment, friends) encourages me to focus on making choices to enjoy the moment, and to postpone learning and study decisions that are important for me.

Options: 1 2 3 4 5

34. The social context that I live in (family, environment, friends) encourages me to focus my behavioral changes toward living life to the fullest, and to not always be placing limits on myself or setting learning and study hours that keep me from doing what I feel like.

Options: 1 2 3 4 5

35. The social context that I live in (family, environment, friends) encourages me to focus on what I have enjoyed in life, when evaluating my own behaviors, and not on what I have done right by setting limits related to learning and study.

Options: 1 2 3 4 5

36. The social context that I live in (family, environment, friends) helps me enjoy myself to the fullest, since it doesn’t press me to change my learning and study behavior, but rather to do what I feel like, if that makes me happy and live fully.

Options: 1 2 3 4 5
